# Gut microbial signatures in autoimmune hepatitis: unlocking diagnostic and therapeutic potential

**DOI:** 10.1097/MS9.0000000000003709

**Published:** 2025-08-14

**Authors:** Fatima Iftikhar, Aliha Iftikhar, Maliha Khalid, Muhammad Talha, Aminath Waafira

**Affiliations:** a Department of Medicine, Liaquat National Hospital and Medical College, Karachi, Pakistan; b Department of Medicine, Jinnah Sindh Medical University, Karachi, Pakistan; c Department of Medicine, King Edward Medical University, Lahore, Pakistan; d School of Medicine, The Maldives National University, Malé, Maldives

**Keywords:** autoimmune hepatitis, dysbiosis, gut microbiota, microbial biomarkers

## Abstract

Autoimmune hepatitis (AIH) is a chronic inflammatory liver disease with unclear etiology but likely results from a complex interplay of genetic susceptibility and environmental triggers. Emerging evidence highlights the role of gut microbiota in AIH pathogenesis, with specific genera such as Veillonella, Lactobacillus, and Oscillospira demonstrating diagnostic value. Dysbiosis-associated biomarkers like lipopolysaccharide and aspartate aminotransferase further support a microbial role in disease onset and progression. Despite these promising developments, rare AIH variants remain poorly characterized due to methodological and population-level limitations. Moving forward, large-scale, longitudinal studies integrating metagenomics, metabolomics, and host genomic data are needed to establish subtype-specific microbial markers and assess the efficacy of targeted interventions such as probiotics and bacteriophage therapy.

*To the Editor*,

Autoimmune hepatitis (AIH) is a rare chronic liver disease with a strong predisposition for middle-aged women. Some individuals carry rare genetic variants that increase vulnerability, although the exact cause remains unclear. Current pathogenetic understanding suggests that genetic susceptibility, when combined with environmental or viral triggers, can initiate the disease. This results in a loss of immune tolerance to liver antigens, leading to chronic liver inflammation. If left untreated, it can progress to cirrhosis, liver failure, or death^[1]^. Early diagnosis and timely intervention are crucial to improving patient outcomes. Exploring microbiome-based biomarkers offers a promising avenue for earlier, noninvasive detection. This approach may enhance diagnostic precision and inform targeted therapeutic strategies in the management of AIH. This article is in line with the TITAN Guidelines on the need for transparency in AI use in healthcare^[2]^.

Moreover, with ongoing scientific and technological progress, there is growing awareness of the critical role played by gut microbiota in the pathogenesis of AIH. Prior research in both human and animal studies has provided evidence linking gut microbiota to the development of AIH^[3]^.

Changes in microbiota diversity and composition may play a crucial role in the early identification and distinction of AIH patients from healthy individuals. Using modeling and cohort validation, a combination of Veillonella, Lactobacillus, Oscillospira, and Clostridium effectively distinguished AIH cases. These results indicate strong predictive potential, supporting improved patient early assessment and informed clinical management decisions^[4,5]^.

Furthermore, the literature suggests that the human microbiome is involved in the development of immune-mediated diseases, such as AIH, and a shift in the intestinal microbiome may contribute to hepatic damage. According to the current literature, there are specific biomarkers produced by the microbiome, the positivity of which indicates a strong relationship with AIH. These biomarkers include plasma lipopolysaccharide, which increases due to dysbiosis in AIH, and serum aspartate aminotransferase as a result of the abundance of Veillonella^[5,6]^. This suggests that such biomarkers can serve as a noninvasive tool to aid in establishing a diagnosis for AIH.

Developing reliable microbiome-based biomarkers for rare AIH variants comes with its challenges. These include heterogeneous cohorts and confounding factors like variation in diet, geographical location, ethnicity, and medication use (e.g. antibiotics). Another challenge arises in the form of small sample sizes due to rare AIH variants, as they are infrequent clinically, making it difficult to assemble adequately powered cohorts and validate subtype-specific microbial signatures. There is also methodological variability across studies, such as differences in sampling approach (mucosal vs stool biopsy), and sequencing platforms (shotgun metagenomics vs 16S rRNA)^[7]^.

In conclusion, gathering evidence demonstrates that gut microbial dysbiosis offers promising diagnostic and mechanistic insights into classic AIH subtypes. However, rare AIH variants remain mostly unexplored. Looking forward, future investigations should prioritize multi-center, longitudinal cohort studies, integrating metagenomic, metabolomic, host-genomic, and mucosal microbiome profiling to uncover subtype-specific marker signatures and causality. Functional interventions, which range from targeted probiotics/symbiotics to bacteriophage therapy, should be studied within to assess safety, efficacy, and biomarker responsiveness through carefully stratified cohorts.

## Data Availability

No data were generated for this manuscript.
